# RASSF1A Signaling in the Heart: Novel Functions beyond Tumor Suppression

**DOI:** 10.1155/2012/154283

**Published:** 2012-04-10

**Authors:** Dominic P. Del Re, Junichi Sadoshima

**Affiliations:** Cardiovascular Research Institute, Department of Cell Biology and Molecular Medicine, UMDNJ-New Jersey Medical School, 185 South Orange Avenue, MSB G-609, Newark, NJ 07103-2714, USA

## Abstract

The RASSF proteins are a family of polypeptides, each containing a conserved Ras association domain, suggesting that these scaffold proteins may be effectors of activated Ras or Ras-related small GTPases. RASSF proteins are characterized by their ability to inhibit cell growth and proliferation while promoting cell death. RASSF1 isoform A is an established tumor suppressor and is frequently silenced in a variety of tumors and human cancer cell lines. However, our understanding of its function in terminally differentiated cell types, such as cardiac myocytes, is relatively nascent. Herein, we review the role of RASSF1A in cardiac physiology and disease and highlight signaling pathways that mediate its function.

## 1. Introduction

The Ras association domain family (RASSF) consists of 10 members: RASSF1-10. Additionally, splice variants of RASSF1, 5 and 6 have been identified [1]. Importantly, all isoforms contain a Ras association (RA) domain either in their C-terminal (RASSF1-6) or N-terminal (RASSF7-10) regions [2]. To date, no known catalytic activity has been described for this family, and the general consensus supposes that RASSF proteins function as scaffolds to localize signaling in the cell. Accordingly, protein-protein interactions are critical in mediating their biological functions. RASSF1 isoform A (RASSF1A) is the most characterized member of the RASSF family. This paper will focus primarily on RASSF1A and its role in cardiovascular biology.

## 2. RASSF1A

RASSF1A was first identified and described by Dammann et al. in 2000 [3]. The *RASSF1 *gene encodes multiple splice variants, including the two predominant isoforms, RASSF1A and C. The RASSF1A isoform is the longest variant of the *RASSF1* gene. Structurally, RASSF1A is a product of exons 1*α*, 2*α*/*β*, 3, 4, 5, and 6, while RASSF1C consists of exons 2*γ*, 3, 4, 5, and 6. Both isoforms contain a C-terminal RA domain; however, RASSF1A has an additional C1 domain that is not present in RASSF1C.

The *RASSF1* gene is located on Chr3p21.3 [3]. This short arm of chromosome 3 is known to exhibit loss of heterozygosity in many tumor models and is thought to harbor tumor suppressor genes. As the literature has shown, RASSF1A fits this description. The *RASSF1A* promoter contains a CpG island that shows a high frequency of hypermethylation in tumors, thereby silencing RASSF1A expression in many human cancers including lung, breast, ovarian, renal, and bladder [4–7]. RASSF1A expression is also lost in numerous cancer cell lines, while RASSF1C expression is seemingly unaffected [4]. Interestingly, recent work suggests that RASSF1C may actually promote tumor progression [8, 9], further distinguishing these two splice variants.

All RASSF proteins have an RA domain, which is thought to necessitate their binding to activated, GTP-bound Ras proteins. While RASSF5 (Nore1) is thought to bind Ras directly, whether RASSF1A is able to associate with Ras is less clear. It has been shown that RASSF1A binds K-Ras *in vitro* [10], and an interaction between ectopically expressed RASSF1A and activated K-Ras has been observed in HEK293 cells [11, 12]. However, other work has found that this interaction only occurs in the presence of Nore1, arguing for an indirect association [13]. Importantly, to our knowledge, there are no reports demonstrating the interaction of endogenous RASSF1A and Ras proteins.

 RASSF1A has several key biological functions typical of tumor suppressor proteins. It has been implicated in the negative regulation of cell cycle progression, cell proliferation, and cell survival [2]. RASSF1A has been shown to localize to microtubules of proliferating cells, increasing microtubule stability and inhibiting cell division [14, 15]. This may be mediated through direct binding or though interaction with microtubule-associated proteins such as C19ORF5 [16]. RASSF1A has also been shown to inhibit proliferation by inhibiting the accumulation of cyclin D1 and arresting cell division [17, 18].

 RASSF1A also promotes apoptosis, which can reportedly occur through multiple mechanisms and is likely cell-type dependent. One mechanism that mediates the apoptotic function of RASSF1A involves protein interaction with modulator of apoptosis-1 (MOAP-1 or MAP-1) [19]. MOAP-1 is normally sequestered in an inactive form in healthy cells. Upon death receptor stimulation, RASSF1A binds MOAP-1, causing its activation and subsequent association with Bax, which leads to apoptosis [19]. Previous work has also demonstrated enhancement of RASSF1A/Mst-mediated cell death by the scaffold CNK1 [20].

### 2.1. RASSF1A and Hippo Signaling

RASSF1A can also elicit inhibitory effects on growth and survival through engagement of the Hippo pathway. The Hippo signaling pathway is a highly conserved kinase cascade that was originally discovered in Drosophila and has been shown to be a critical regulator of cell proliferation, survival, and organ growth [21]. Three members of this pathway, dRASSF, Salvador and Hippo, contain the SARAH (Salvador-RASSF-Hippo) domain, which is conserved in its mammalian counterparts RASSF1-6, WW45, and Mst1/2, respectively [22]. The SARAH domain is critical for homo- and heterodimerization between components [23–27]. While the Drosophila ortholog dRASSF is known to antagonize Hippo activation in the fly [28], it has been demonstrated that RASSF1A promotes phosphorylation and activation of Mst 1/2 by inhibiting the phosphatase PP2A in mammalian systems [29, 30].

The biological relevance of RASSF1A-mediated activation of Hippo signaling has also been investigated. Matallanas et al. reported a RASSF1A-Mst2-Yap-p73-PUMA signaling axis that promotes apoptosis in mammalian cells [31]. Hippo signaling is also important for maintaining intestinal homeostasis and tissue regeneration in response to injury. Mouse models with conditional disruption of either Mst1/2 or Sav1 in the intestinal epithelium displayed hyperactivation of Yes-associated protein (Yap), increased intestinal stem cell (ISC) proliferation, and increased polyp formation following dextran sodium sulfate (DSS) treatment [32, 33]. Similarly, loss-of-function mutations of Hippo components in the fly midgut caused increased ISC proliferation [34]. These findings suggest that perhaps Hippo signaling serves a more global role in regulating organ integrity, structure, and response to injury, and that perturbation of this pathway can lead to aberrant growth and dysfunction.

## 3. Cardiovascular Function of RASSF1A

In 2005, two independent groups generated and published findings regarding the systemic deletion of the *Rassf1a* gene variant in mice [35, 36]. Both described similar phenotypes involving the spontaneous generation of tumors, particularly in aged mice, thus further supporting the notion that RASSF1A is a bona fide tumor suppressor [35, 36]. Not surprisingly, nearly all studies involving RASSF1A to date are related to cancer biology with few reports related to the cardiovascular field.

RASSF1A is ubiquitously expressed and has been detected in heart tissue [3, 37, 38]. Initial investigation into the role of *Rassf1* gene products in a cardiac context came from the Neyses laboratory [39]. Their findings demonstrated that both RASSF1A and RASSF1C could associate with the sarcolemmal calcium pump, PMCA4b, in neonatal rat cardiac myocytes. This interaction was shown to mediate the inhibition of ERK, and subsequent Elk transcription and suggested the possibility that RASSF1A could modulate cardiac myocyte growth [39].

### 3.1. **Rassf*1*a*^−/−^* Mice

 Five years later, the same group demonstrated that RASSF1A does in fact negatively regulate cardiac hypertrophy *in vivo* using *Rassf*1*a* ^−/−^ mice [37]. Although these mice have increased susceptibility to spontaneous tumorigenesis [36], no apparent cardiovascular phenotype was observed under basal conditions, that is, no differences in heart size, morphology, or function compared to WT. However, when *Rassf*1*a* ^−/−^ mice were challenged with pressure overload, they responded with an exaggerated hypertrophic response, evidenced by significantly greater increases in heart weight/body weight and hypertrophic gene expression (ANP, BNP, *β*-MHC). Cardiac myocytes of *Rassf*1*a* ^−/−^ mice were significantly larger, which explains the augmented heart growth. Chamber dilation of *Rassf*1*a* ^−/−^ mouse hearts was observed by echocardiography, consistent with eccentric hypertrophic remodeling. Hemodynamic analysis of WT and *Rassf*1*a* ^−/−^ mice showed a rightward shift in PV loops following pressure overload in *Rassf*1*a* ^−/−^ hearts, yet dP/dt_max⁡_, dP/dt_min⁡_, and fractional shortening were not altered in *Rassf*1*a* ^−/−^ mice compared to WT.

 To examine RASSF1A function in cardiac myocytes, Oceandy et al. utilized a neonatal rat cardiac myocyte (NRCM) culture and the forced expression of RASSF1A through adenoviral gene transfer [37]. Increased RASSF1A expression inhibited phenylephrine-(PE-) induced cardiac myocyte growth and suppressed Raf-1 and ERK1/2 activation by PE treatment. Conversely, both Raf-1 and ERK1/2 phosphorylation were increased in  *Rassf*1*a* ^−/−^ hearts following pressure overload, suggesting negative regulation of MAPK signaling by RASSF1A. Deletion mutants of RASSF1A revealed an important function of the N-terminus of RASSF1A that disrupts the binding of active Ras and Raf-1, thus preventing ERK activation and cardiac myocyte growth.

### 3.2. Cardiac Myocyte-Specific *Rassf1a* Deletion

 To better understand the function of RASSF1A in cardiac myocytes *in vivo*, we crossed genetically altered mice harboring a floxed *Rassf1a *allele [35] with mice harboring the Cre recombinase transgene driven by the *α*-MHC promoter. This strategy disrupted endogenous *Rassf1a* gene expression and ensured cardiac myocyte specificity [38, 40]. Similar to the *Rassf*1*a* ^−/−^  mice, *Rassf1a^F/F^-Cre* mice had no obvious baseline cardiac phenotype. Although we also found exaggerated heart growth in the *Rassf*1*a* ^−/−^ mice in response to pressure overload, the *Rassf1a^F/F^-Cre* mice unexpectedly had attenuated hypertrophy, that is, smaller hearts and cardiac myocytes, compared to *Rassf1a^F/F^* and *α*-MHC-Cre controls [38]. Furthermore, *Rassf1a^F/F^-Cre* mice had significantly less fibrosis and myocyte apoptosis, and better cardiac function following pressure overload. This was in stark contrast to the *Rassf*1*a* ^−/−^ mice, which presented significantly more fibrosis and a decline in cardiac function comparable to the levels found in WT mice.

 As an alternative approach we also generated two different cardiac-specific transgenic mouse lines: the first expressing wild-type RASSF1A and the second expressing a RASSF1A SARAH domain point mutant (L308P) that renders it unable to bind Mst1 [41]. Interestingly, we found that increased RASSF1A expression in the heart caused increases in Mst1 activation, cardiac myocyte apoptosis, and fibrosis, and led to worsened function following pressure overload. Conversely, RASSF1A L308P TG mice had significant reductions in Mst1 activation, apoptosis and fibrosis, while cardiac function was preserved after stress [38]. These opposing phenotypes strongly implicate Mst1 as a critical effector of RASSF1A-mediated myocardial dysfunction.

**Figure 1:**
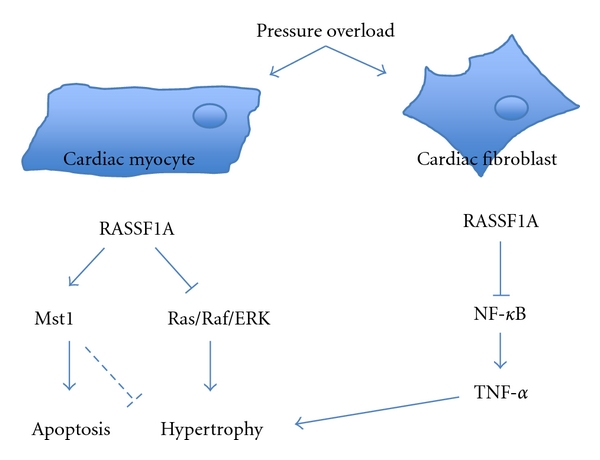
In cardiac myocytes, RASSF1A can prevent hypertrophy through disruption of Ras/Raf-1/ERK MAPK signaling. RASSF1A can also activate Mst1 to elicit apoptosis. In cardiac fibroblasts, RASSF1A represses NF-*κ*B transcriptional activity and inhibits TNF-*α* production and secretion, thereby preventing paracrine-mediated hypertrophic signaling between fibroblast and myocyte.

 In cultured NRCMs, increased RASSF1A expression elicited activation of Mst1 and caused Mst1-mediated apoptosis. However, in primary rat cardiac fibroblasts, RASSF1A had a more pronounced effect on inhibition of cell proliferation rather than survival. Indeed, we found that silencing of RASSF1A in fibroblasts caused increased cell proliferation. Additionally, RASSF1A depletion led to an upregulation of NF-*κ*B-dependent TNF-*α* expression and secretion in cardiac fibroblasts, while no change in IL-1*β*, IL-6, or TGF-*β*1 was observed. Through conditioned medium transfer experiments, we demonstrated that TNF-*α* secretion from fibroblasts promotes cardiac myocyte growth. Furthermore, treatment of *Rassf1a^−/−^* mice with a neutralizing antibody against TNF-*α* was able to rescue the augmented heart growth and fibrosis observed following pressure overload [38]. These data strongly implicated TNF-*α* as a critical paracrine factor influencing the cardiac myocyte growth response to stress *in vivo*. This work also demonstrated the cell-type specificity of RASSF1A signaling in the heart and highlighted a novel signaling pathway downstream of RASSF1A/Mst1 that mediates a paracrine effect *in vivo* (see Figure 1). This mechanism involving multiple cell types, and paracrine signaling among them is rather unique and contrasts with more established signaling paradigms of cardiac hypertrophy including calcineurin/NFAT, HDAC/MEF2 and MEK/ERK pathways, which have been elucidated in the cardiac myocyte [42].

### 3.3. Hippo Signaling in the Heart

 Our previous work has demonstrated the functional importance of Hippo signaling in the heart. Using genetically altered mouse models we showed that increased expression of Mst1, and subsequent activation of the Hippo pathway, caused increased apoptosis, dilated cardiomyopathy, and premature death [43]. Interestingly, expression of Mst1 also attenuated cardiac myocyte hypertrophy thereby impairing the heart's ability to appropriately respond to stress. In contrast, expression of a kinase-inactive Mst1 mutant (DN-Mst1) prevented cell death and protected the heart from insult [43]. Lats1/2 kinases (mammalian homologs of Warts) are targets of Mst1/2 that can phosphorylate and inactivate Yap, thereby inhibiting Yap-mediated gene transcription [44]. Similar to our findings related to Mst1, we demonstrated that transgenic expression of Lats2 in the heart led to inhibited growth and worsened function [45]. Conversely, kinase-inactive Lats2 (DN-Lats2) transgenic mice had larger hearts both at baseline and following pressure overload and displayed attenuated cardiac myocyte apoptosis in response to stress [45]. Taken together, these results provide further evidence that activation of Hippo signaling, via increased Mst1 or Lats2 expression, inhibits cardiac myocyte growth and promotes apoptosis in the adult heart. Furthermore, selective inhibition of Hippo signaling in the cardiac myocyte (DN-Mst1 or DN-Lats2 TG) confers protection against insult, similar to what we observed in the cardiac myocyte-specific RASSF1A deleted mice [38]. However, the hypertrophic response in these two models was opposite, which may result from a Hippo-independent pathway(s) downstream of RASSF1A. It should be pointed out that studies of adult mouse models using cardiac myocyte-restricted deletion of Mst1/2, Lats1/2 or Yap have not been published. Findings from these models should be helpful in further elucidating the role of Hippo signaling components in the adult murine heart.

Recent work from the Martin laboratory demonstrated the importance of mammalian Hippo signaling during cardiac development and cardiac myocyte proliferation [46]. Conditional deletion of Salvador (Sav1) in the embryonic heart, driven by Nkx2.5-Cre expression, caused increased myocyte proliferation and cardiac enlargement and was mediated by hyperactivation of Yap and subsequent Wnt/*β*-catenin-regulated gene expression. In a similar vein, direct targeting of Yap expression in the developing mouse heart further demonstrated its role in governing both myocyte proliferation and heart growth [47]. Interestingly, both reports described an interaction between Yap and Wnt signaling, highlighting additional Hippo signaling crosstalk in the heart.

## 4. Conclusion

 Fueled by the initial reports described herein, investigation into the role of RASSF1A in cardiovascular biology has begun to accelerate. Yet many questions remain outstanding. Among them, what are the upstream inputs that regulate RASSF1A function? What is the mechanism responsible for RASSF1A cell-type-specific signaling? What are the molecular constituents of the RASSF1A complex? Does RASSF1A have additional Mst1-independent functions in the heart, as has been demonstrated in tumor cell lines [41]? Recent work identified activated K-Ras as a promoter of RASSF1A signaling in colorectal cancer cells [48]. This finding begs the question of whether K-Ras or additional Ras isoforms regulate RASSF1A in other systems and cell types. Based on our findings in *Rassf1a*-deleted mice [38], we speculate that the difference in proliferative capacity between cardiac myocytes and fibroblasts may explain the distinct effects of RASSF1A signaling in the heart. There may also be differences in the expression or localization of signaling components, thereby modulating their ability to effectively signal in certain cell types. Exposure to diverse signals and cues in the extracellular milieu may also contribute to varied outcomes downstream RASSF1A.

As we continue to elucidate the role of RASSF1A and Hippo signaling in the heart, its importance in cardiac development, physiology, and disease is becoming apparent. Of course, translating these findings into meaningful therapeutic strategies remains the greatest challenge. Our work has shed light on the importance of cell type specificity RASSF1A in determining pathological outcomes [38]. We also defined a paracrine mechanism functioning downstream of RASSF1A in response to cardiac stress [38]. It is likely that additional complexities remain to be uncovered and will ultimately influence possible interventions to manipulate RASSF1A and treat heart disease.

RASSF1A signaling is diverse and our knowledge regarding RASSF1A function is rapidly expanding. Given that a bridge from cancer to cardiovascular biology is in place, it is likely that as additional RASSF1A mechanisms of action are discovered, its impact on cardiac biology will continue to grow.
